# Multi-Timepoint Metabolic Fingerprinting of a Post-Episode Period of Hypoglycemia and Ketoacidosis Among Children With Type 1 Diabetes

**DOI:** 10.3389/fmolb.2022.869116

**Published:** 2022-06-23

**Authors:** Beata Małachowska, Karolina Pietrowska, Wojciech Młynarski, Agnieszka Szadkowska, Adam Krętowski, Michał Ciborowski, Wojciech Fendler

**Affiliations:** ^1^ Medical University of Lodz, Department of Biostatistics and Translational Medicine, Łódź, Poland; ^2^ Medical University of Bialystok, Clinical Research Center, Metabolomics Laboratory, Białystok, Poland; ^3^ Medical University of Lodz, Department of Pediatric Oncology and Hematology, Łódź, Poland; ^4^ Medical University of Lodz, Department of Pediatrics, Diabetology Endocrinology and Nephrology, Łódź, Poland; ^5^ Medical University of Bialystok, Department of Endocrinology, Diabetology and Internal Medicine, Bialystok, Poland; ^6^ Dana-Farber Cancer Institute, Department of Radiation Oncology, Boston, MA, United States

**Keywords:** diabetes ketoacidosis (DKA), hypoglycemia, LC-MS, metabolomics, serum, type 1 diabetes

## Abstract

**Background:** Acute complications of type 1 diabetes mellitus such as diabetes ketoacidosis (DKA) and hypoglycemia (HG) are detrimental in a short- and long-term perspective. Restoration of normoglycemia and correction of pH do not mean that all metabolic disturbances caused by HG or DKA are immediately reversed.

**Aim:** This study aimed to identify serum metabolic changes caused by an episode of DKA and HG that may indicate the mechanisms contributing to long-term consequences of DKA/HG.

**Materials and methods:** Four groups of children with type 1 diabetes were recruited. The first two study groups included patients after an episode of DKA or HG, respectively. Additionally, two comparative groups were recruited—children with established type 1 diabetes (EDM) and patients with newly diagnosed diabetes without diabetes ketoacidosis (NDM). Serum samples were collected in three group-specific time points (since the hospital admission): HG 0h-12h–48h; DKA or NDM 0h-24h–72 h; and one random fasting sample from patients with EDM. Two batches of 100 samples each were created: for DKA batch 20 × 3 DKA patients, 10 × 3 NDM and 10 EDM; for HG batch: 10 × 3 HG patients, 25 EDM and 15 × 3 NDM. All patients within the batches were age and sex matched. Metabolic fingerprinting was performed with LC-QTOF-MS.

**Results:** Four metabolites were associated with a DKA episode occurring in the preceding 72 h: three were found higher after the DKA episode versus comparative groups: lysophosphatidylcholine (LPC) (18:1), sphingomyelins (SM) (34:0 and d18:0/15:0), and one was found lower: LPC (18:0). Similarly, four metabolites were identified for the HG episode in the last 48 h: three were found higher after the HG episode versus comparative groups: two lysophosphatidylethanolamines (LPE) (18:2 and 20:3) and one LPC (18:2); and one was found lower after the HG episode: oxy-phosphatidylocholine (PC O-34:4).

**Conclusions:** We found eight metabolites whose levels may be traced in the serum, indicating the DKA or HG episode for up to 72 h and 48 h, respectively. Acute complications of diabetes may cause persistent metabolic disturbances long after pH and glucose level normalization.

## 1 Introduction

Diabetes ketoacidosis (DKA) and hypoglycemia (HG) are typical acute complications of type 1 diabetes. The key challenge for patients with type 1 diabetes, their guardians, and healthcare personnel is to identify DKA/HG risk factors, predict their imminent onset, and counteract them rapidly.

DKA most commonly accompanies newly diagnosed type 1 diabetes and typically affects 15%–70% of new diabetic cases (Wolfsdorf et al., 2018), with substantial variability between countries. Among patients with established type 1 diabetes (EDM), DKA occurs with a frequency of 1–10 episodes/100 patients per year. The most serious aftermath of DKA is brain oedema (BO). Clinical symptoms of BO are present in 0.5–0.9% of DKA cases, but impaired consciousness appears in 4–15% and is associated with BO features observed by neuroimaging (Glaser et al., 2006; Glaser et al., 2008). Regardless of BO, every episode of DKA should be considered a life-threatening condition (Cameron et al., 2014). DKA is the main cause of death among children with type 1 diabetes younger than 15 years of age (Morgan et al., 2018). DKA is accompanied by dehydration which is typically corrected within 12 h; however, fluid therapy is maintained for 24–48 h (Fiordalisi et al., 2007). There is much evidence that DKA episodes disturb metabolism and neurological functions for an extended period of time, resulting in severe neurological sequelae in 35% of DKA with BO survivors (Edge et al., 2001). Additionally, patients with DKA at the onset of diabetes show a further worse metabolic control of diabetes in comparison to patients without DKA (Duca et al., 2017; Shalitin et al., 2018).

HG is the most common acute diabetes complication (Kapoor, 2016) for established type 1 diabetes. Among adults, severe HG is defined as an episode requiring the help of others. In children, most episodes require assistance, so for this population, seizures or loss of consciousness define severe HG (Clarke et al., 2009). The frequency of severe HG is estimated at 5–20 episodes per 100 patient-years (Ly et al., 2014). The risk of HG is the main limiting factor for achieving good metabolic control and remains the heaviest psychological burden for type 1 diabetic patients and their families (Abraham et al., 2018). Each HG episode may exert a lasting negative effect on the patient’s neurological functions, e.g., spatial long-term memory performance (Hershey et al., 2005), cognition (Asvold et al., 2010; Rovet and Ehrlich, 1999), and vestibular organ function impairment (Gawron et al., 2002). Also, metabolic aftermaths are common and include: reactive hyperglycemia (Clayman, 1970), increased insulin resistance (Lucidi et al., 2010), increased pro-inflammatory state (Kiec-Wilk et al., 2016), and increasing hypoglycemia unawareness (Peczyńska et al., 2004).

The aim of this study was to identify serum metabolic changes caused by an episode of DKA and HG, that can be detected despite normalization of parameters typically changed during the episodes and that may indicate on mechanisms contributing to long-term consequences of DKA/HG episodes.

## 2 Materials and Methods

### 2.1 Patient Recruitment

The study was approved by the Institutional Bioethical Committee (RNN/71/14/KE) and patient recruitment was performed between 30.10.2014 and 07.10.2016 in the Department of Pediatrics, Diabetology, Endocrinology and Nephrology of the Medical University of Lodz. The study protocol and its scientific purposes were explained to the patients and their legal guardians. Patients meeting the inclusion criteria were asked to participate in the study. Written consent to participate in the study was obtained from the patients and/or their legal guardians. We prospectively recruited patients who met the following inclusion criteria: age between 2 and 19 years old; diagnosis of type 1 diabetes according to ISPAD 2014 criteria (Rubio-Cabezas et al., 2014); lack of known concomitant genetic disorders; and diabetes treatment with insulin alone. Additionally, group-specific inclusion criteria were as follows: for the HG group—glucose concentration below 70 mg/dl during the episode and being unconscious or seizures; for DKA group—pH < 7.30 and HCO_3_^−^ <15 mmol/L at diabetes diagnosis; for NDM group—pH > 7.35 and HCO_3_^−^ >21 mmol/L at diabetes diagnosis; and for EDM group—diabetes duration above 6 months and lack of DKA or HG episodes within last 3 months. Clinical data were collected from the hospital information system. All patients within the batches were age- and sex-matched.

### 2.2 Sample Collection

**FIGURE 1:**
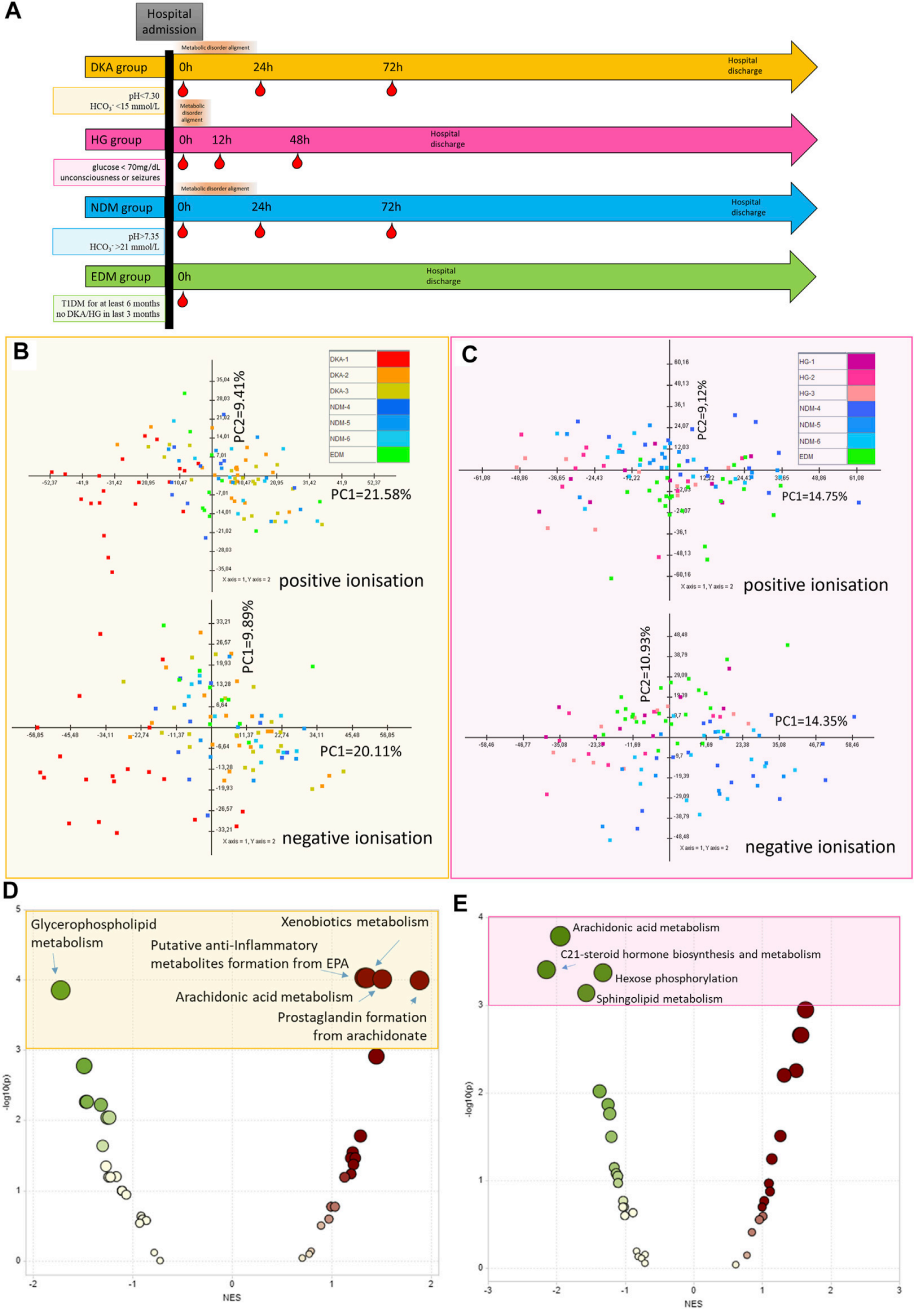
Protocol for sample collection for each study group (DKA, diabetes ketoacidosis, HG, hypoglycemia) and each comparative group (NDM, new onset of diabetes mellitus with DKA and EDM, established diabetes mellitus). Red droplets indicate the time points of blood withdrawal **(A)**. The principal component analysis of metabolic features passing technical data filtering for the DKA batch **(B)** and HG batch **(C)**. Metabolite Set Enrichment Analysis results based on results from the DKA batch (using metabolites from combined comparison between DKA-2&DKA-3 and NDM-4&NDM-5 groups with *p* < 0.15) **(D)** and the HG batch (using metabolites from combined comparison between HG-1&HG-2&HG-3 and EDM with *p* < 0.15) **(E)**.

The sample collection protocol is shown in Figure 1A. For the HG group, the first sample was collected right after hospital admission (0 h), the second sample was collected 12 h after the admission, and the third sample was collected 48 h after the admission. For the DKA and NDM groups, the first samples were collected at hospital admission (0 h), the second—after 24 h admission, and the third ones—after 72 h after admission. Different protocols of the sample collection for HG and DKA/NDM patients arose from different dynamics of metabolic disturbances typical for the evaluated groups and the fact that HG patients were admitted after the episode and DKA/NDM were admitted with an ongoing disturbance and different hospitalization time: 2–3 days for the HG group and up to 2 weeks for the DKA–NDM group. Also, a limiting factor was the availability of an intravenous line in patients which is usually kept up to 3 days. For the EDM group only one sample was collected during routinely performed control laboratory tests as we did not assume a dynamic change in their metabolomic profile. Due to ethical issues, blood samples were collected only from already installed intravenous catheter or during routinely performed blood collections. Each time, a sample of 4 ml was collected from the patient to the vial with a clot activator (silica particles) (Becton Dickinson, New Jersey, NJ, United States). In order to standardize the sample processing protocol, we used vials from the same manufacturer (series 8516328 BD/REF 369032/2015–08/4113194). Between 60 and 120 min after the blood collection, serum isolation was performed by centrifugation for 10 min with 800 g. After serum separation, the biological material was frozen in -80°C degree until metabolic fingerprinting.

### 2.3 Serum Sample Preparation Before Metabolomics Fingerprinting

On the day of the metabolomic analysis, samples were thawed on ice. Protein precipitation and metabolite extraction were performed by vortex-mixing (for 1 min) one volume of the serum sample with four volumes of freeze cold (–20°C) methanol/ethanol (1:1) mixture containing 1 ppm of zomepirac (IS). After extraction, the samples were stored on ice for 10 min and centrifuged at 21,000 × g for 20 min at 4°C. The supernatant was filtered through a 0.22-µm nylon filter into glass vials.

Quality control (QC) samples were prepared by mixing an equal volume of all samples. The obtained mixture was prepared following the same procedure as the rest of the samples. QC samples were used at the beginning of the analysis (10 injections), in order to obtain the system stability, and then after every nine samples, in order to control the system stability and productiveness of the sample measurements.

### 2.4 Used Chemicals and Reagents

Blank samples included mixtures of methanol/ethanol (1:1) that were prepared in the same way as biological samples. Zomepirac sodium salt (used as the internal standard (IS), arachidonic acid, docosahexaenoic acid, lysophosphatidylethanolamine (LPE) 18:0, phosphatidylethanolamine (PE) 16:0/22:6, LS-MS-grade acetonitrile, methanol, LC-grade formic acid, and ethanol were purchased from Sigma-Aldrich Chemie GmbH (Steinheim, Germany). The API-TOF reference mass solution kit (G1969-850001) and tuning solutions, ESI-L low-concentration tuning mix (G1969-85000), and ESI-TOF Biopolymer Analysis reference masses (G1969-850003) were purchased from Agilent Technologies (Santa Clara, CA, United States). Purified water was obtained using the Milli-Q Integral three system (Millipore SAS, Molsheim, France) in order to prepare solution A.

### 2.5 Metabolic Fingerprinting

Metabolic fingerprinting was performed using liquid chromatography coupled to the tandem (quadrupole and time of flight, Q-TOF) mass spectrometry (LC-MS) system following the previously described protocol (Daniluk et al., 2019). Samples from the HG and DKA groups were analyzed in the separate batches to ensure data quality.

Analyses were performed in compliance with the current standards in force for the metabolomic measurements based on mass spectrometry and quality control methodology (Dunn et al., 2011; Godzien et al., 2015a).

Samples were randomly analyzed using the LC-MS system consisting of 1290 Infinity LC with a degasser, two binary pumps, and a thermostated (4°C) autosampler coupled to a 6550 iFunnel Q-TOF-MS detector (both Agilent Technologies, Santa Clara, CA, United States). Analyses were performed in positive (ESI+) and negative (ESI-) ion modes, whereby 1 µL of the sample was injected into a thermostated (60°C) Zorbax Extend- C18 RRHT (2.1 × 50 mm, 1.8-µm particle size, Agilent Technologies) chromatographic column. The flow rate was 0.6 ml/min with solvent A (water with 0.1% formic acid) and solvent B (acetonitrile with 0.1% formic acid). The chromatographic gradient started at 5% of phase B for the first minute, followed by an increase of phase B to 80% (from 1 to 7 min) and to 100% (from 7 to 11.5 min). After reaching 100%, the gradient returned to their initial conditions (5% phase B) in 0.5 min, which were kept from 12 to 15 min.

The mass spectrometer operated in the full scan mode from 50 to 1,000 mass (m/z). The capillary voltage was set to 3,000 V for the positive and 4,000 V for the negative ionization mode. Nozzle voltage was 1,000 V. The drying gas flow rate was 12 L/min at 250°C and gas nebulizer at 52 psig; the fragmentor voltage was 250 V for the positive and negative ionization modes. Data were collected in the centroid mode at a scan rate of 1.5 scan per second. Accurate mass measurements were obtained by means of a calibrant solution delivery using a dual-nebulizer ESI source. A calibrating solution containing reference masses at m/z 121.0509 (protonated purine) and m/z 922.0098 (protonated hexakis [(1H,1H,3H-tetrafluoropropoxy) phosphazine or HP-921] in the positive ion mode or m/z 119.0363 (proton abstracted purine) and m/z 966.0007 (formate adduct of HP-921) in the negative ion mode was continuously introduced by an isocratic pump (Agilent, Santa Clara, CA, United States) at a flow rate of 0.5 ml/min (1:100 split).

### 2.6 Lecithin-Cholesterol-MS Data Curation

The raw data collected by the analytical instrumentation were cleaned of background noise and unrelated ions by the molecular feature extraction (MFE) tool in the Mass Hunter Qualitative Analysis Software (B.07.00, Agilent, Santa Clara, CA, United States). The MFE creates a list of all possible components described by mass, retention time (RT), and abundance.

The limit for the background noise for data extraction by MFE was set to 1,500 and 1,000 counts for the positive and negative ion modes, respectively. To identify co-eluting adducts of the same feature, the following adduct settings were applied: + H, + Na, and + K in the positive ion mode and −H, + HCOO, and + Cl for the negative ion mode. Dehydration neutral losses were also allowed in both ionization modes. Sample alignment and data filtering were performed using Mass Profiler Professional 12.6.1 (Agilent, Santa Clara, CA, United States). Parameters applied for the alignment were 1% for RT and 15 ppm for the mass variation. In the quality assurance (QA) procedure, metabolic features detected in >80% in QC samples with the coefficient of variation (CV) < 20% were kept for further data treatment.

### 2.7 Metabolite Identification

Accurate masses of features were searched against the METLIN (http://metlin.scripps.edu), KEGG (https://www.genome.jp/kegg/pathway.html), LIPIDMAPS (http://lipidmaps.org), and HMDB databases (http://hmdb.ca), which were simultaneously accessed by a CEU Mass Mediator (http://ceumass.jpg.uspceu.es/mediator/). The identity of the metabolites was confirmed by matching the experimental MS/MS spectra to the MS/MS spectra from databases or fragmentation spectra and retention time obtained for the metabolite’s standard. Experiments were repeated with identical chromatographic conditions to the primary analysis. Ions were targeted for collision-induced dissociation (CID) fragmentation on the fly based on previously determined accurate mass and retention time. Phospholipids were identified based on a previously described characteristic fragmentation pattern (Godzien et al., 2015b). Details on metabolite identification are provided in Supplementary Table S1. Internal standard verification of two metabolites for which IS was commercially available is shown in Supplementary Table S2.

### 2.8 Statistical Data Analysis

A quality assurance procedure was performed and the metabolomic features with RSD (relative standard deviation) for QC samples ≥20% or detectable in more than 1/3 blank samples were filtered out. Due to the high variance of a total signal obtained from each sample (probably due to samples dilutions caused by ongoing fluid therapy of the patients), the levels of themetabolomic features were divided by the sample total signal and multiplied by 100,000,000. This should make the metabolomic feature levels more comparable between samples with different dilutions. One sample from the NDM group (X62-5—second time point) was found to be an outlier in the negative ionization mode in the HG batch due to very low total signal, and most metabolic features undetectable—probably due to a problem with injection to the analytical system (Supplementary Figure. S1). It was excluded from further analysis.

For the statistical analysis, metabolomic features occurring in at least 80% of samples from each group were selected. A univariate comparison was performed with ANOVA and Benjamini–Hochberg for multiple comparison correction. For the DKA series, 48h and 72 h samples were compared with the NDM group samples from respective time points. DKA samples from the first time point were omitted here due to an ongoing DKA state that greatly affected the results of this analysis. For HG series, all time point samples were used to compare them with the EDM group. In this analysis, each time point was kept as a separate group decreasing statistical power thus for the *post-hoc* analysis, features with FDR<0.15 were selected. A Bonferroni-adjusted *t*-test was used for *post-hoc* pairwise comparisons. In the univariate analysis, for the DKA batch, the NDM group was used as control, while for the HG batch, the EDM group was used as control.

An advanced statistical analysis was performed with the Metaboanalyst 4.0 tool (Chong et al., 2018) with data filtered in the same way as for a simple statistical analysis. Additionally, missing data were filled with the *K-*NN (nearest neighbor) method and were scaled with the Pareto algorithm. An MS peak to Pathway analysis was performed with the GSEA algorithm and with the MFN database. Two OPLS-DA (orthogonal partial least square discriminant analysis) models were built for each study group. The first one was to discriminate between study groups from EDM samples, and the second one to discriminate from all NDM samples. Metabolomic features meeting the following criteria: p[1]>|0.4| and p(corr)[1]>|0.2| from both models were selected.

For features meeting the selection criteria in the simple and advanced analyses, an identification attempt was made.

Collected clinical data were compared between the groups by parametric or non-parametric tests based on data distribution and homoscedasticity. Metabolite levels were log-transformed with base 10. Correlations between the metabolite levels and other clinical data were performed with Pearson or Spearman correlation—depending on the variable distribution. Diagnostic utility of identified metabolites was performed with the ROC curve analysis. Sensitivity and specificity along with 95%CI (confidence interval) were calculated with VassarStats (http://vassarstats.net/). A clinical data analysis was carried out with STASTISTICA 13.1 (TIBCO Software, Palo Alto, CA, United States).

## 3 Results

### 3.1 Study Group Characteristics

For the DKA analysis, a batch of samples was collected from: 20 patients hospitalized due to DKA (with three samples collected per patient 0h-24h–72h, N_samples_ = 60), 10 patients with newly diagnosed type 1 diabetes mellitus (NDM, with three samples collected per patient 0h-24h–72h, N_samples_ = 30), and 10 patients with established diabetes mellitus (EDM, one sample collected per patient N_samples_ = 10). For the HG batch, we recruited 10 patients hospitalized after the episode of HG (with three samples collected per patient 0h-12h–48h, N_samples_ = 30), 25 patients with EDM (1 sample collected per patient N_samples_ = 25), and 15 patients with NDM (3 samples collected per patient 0h-24h–72h, N_samples_ = 45). Data collection protocol for each group is shown in Figure 1A. Study group characteristics for the DKA batch is shown in Table 1 and for the HG batch in Table 2.

**TABLE 1:** Study groups’ characteristics selected for metabolomics fingerprinting of the past hypoglycemia episode.

Sex (% males)	HG (N = 10)	NDM (N = 15)	EDM (N = 25)	p
	50%	53.33%	52%	1.0
	Mean ± SD	Mean ± SD	Mean ± SD	
Current age (years)	15.05 ± 2.74$	9.26 ± 3.33*$	15.00 ± 2.58*	**<0.0001 (K-W)**
Age at diabetes onset (years)	9.10 ± 3.52	9.26 ± 3.33	9.76 ± 3.81	0.8553
Duration of diabetes (years)	5.95 ± 3.61$	0.00 ± 0.00*$	5.25 ± 3.55*	**<0.0001 (K-W)**
HbA1c (%)	8.17 ± 2.07$	11.26 ± 1.78*$	7.31 ± 1.02*	**<0.0001 (K-W)**
DDI (U/kg)	0.82 ± 0.16	0.57 ± 0.21	0.77 ± 0.27	**0.0191**
BMI z-score	0.38 ± 1.00	−0.50 ± 1.48	0.37 ± 0.85	**0.0436**
C-peptide (ng/mL)	0.13 ± 0.19$	0.54 ± 0.42$	0.26 ± 0.36	**0.0230**
Total cholesterol (mg/dL)	166.09 ± 31.70	162.81 ± 38.22	164.81 ± 28.23	0.9667
Triglycerides (mg/dL)	75.03 ± 19.98	79.38 ± 37.45	73.24 ± 21.74	0.9510
HDL (mg/dL)	60.91 ± 16.40	49.07 ± 12.34*	62.38 ± 13.60*	**0.0149**
LDL (mg/dL)	94.82 ± 23.46	104.33 ± 42.37	89.28 ± 21.10	0.4271 (Welch)
Hemoglobine (g/dL)	13.77 ± 1.14	13.96 ± 1.00	14.03 ± 1.19	0.8248
RBC (mln/mm^3^)	4.66 ± 0.42	4.95 ± 0.32	4.74 ± 0.37	0.1104
Hematocrit (%)	40.23 ± 3.00	40.57 ± 2.43	41.23 ± 3.32	0.6281
MCHC (g/dL)	34.23 ± 0.80	34.40 ± 1.00	34.04 ± 0.40	0.3900 (Welch)
MCV (fL)	86.60 ± 3.53$	82.20 ± 2.76$*	87.00 ± 2.84*	**<0.0001**
WBC (10^3^/mm^3^)	10.62 ± 3.21#	8.39 ± 2.69*	6.18 ± 1.30#*	**0.0006**
Platelets (10^3^/mm^3^)	251.40 ± 41.96	270.53 ± 89.23	250.20 ± 52.55	0.9831 (K-W)
Na^+^ (mmol/L)	139.05 ± 3.23$	133.80 ± 5.42$	138.23 ± 1.59	**0.0188** (Welch)
K^+^ (mmol/L)	4.86 ± 0.39#	4.57 ± 0.41	4.24 ± 0.65#	**0.0153** (K-W)
eGFR (mL/min/1,73m^2^)	109.05 ± 24.58	88.52 ± 25.14	94.55 ± 15.01	0.0548
pH	7.39 ± 0.03	7.39 ± 0.03	Within normal ranges (7.35−7.45)	0.6903
pCO_2_ (mm Hg)	38.61 ± 3.61$	33.97 ± 4.40$	Within normal ranges (35−45)	**0.0110**
pO_2_ (mm Hg)	70.85 ± 10.68	71.97 ± 12.23	Within normal ranges (75−100)	0.8065 (UMW)
HCO_3_^−^ (mmol/L)	22.81 ± 1.75$	20.93 ± 1.87$	Within normal ranges (21−27)	**0.0191**
O_2_sat. (%)	90.70 ± 12.07	93.23 ± 10.13	Within normal ranges (95−98)	0.2384 (UMW)
BE (mval/l)	−1.41 ± 1.75$	−4.23 ± 2.55$	Within normal ranges (−2.3−2.3)	**0.0036** (UMW)

* significant difference in post-hoc test between NDM, and EDM, #—significant difference in post-hoc test between HG, and EDM, $—significant difference in post-hoc test between HG, and NDM. ANOVA *p*, value test was provided in the table, unless stated otherwise in round bracket: K-W—Kruskal-Wallis test, UMW, Mann-Whitney *U* test, Welch—Welch’s ANOVA, test. DDI, daily dose if insulin; BMI, body mass index; HDL, high density lipoprotein; LDL, low density lipoprotein; RBC, red blood cells; MCHC, mean corpuscular hemoglobin concentration; MCV, mean corpuscular volume; WBC, white blood cells; eGFR, estimated glomerular filtration rate; BE, base excess. Values in boldface are significant at p < 0.05.

**TABLE 2:** Study groups’ characteristics selected for metabolomics fingerprinting of the past HG episode.

Sex (% males)	DKA (N = 20)	NDM (N = 10)	EDM (N = 10)	p
	50%	50%	50%	1.0
	Mean ± SD	Mean ± SD	Mean ± SD	
Current age (years)	10.35 ± 3.54	9.91 ± 3.78	10.41 ± 3.41	0.9383
Age at diabetes onset (years)	8.06 ± 4.25	9.91 ± 3.78*	5.41 ± 2.65*	**0.0388**
Duration of diabetes (years)	2.28± 4.34#	0.0 ± 0.0*	5.00 ± 3.30*#	**0.0001** (K-W)
HbA1c (%)	11.79 ± 2.59#	11.25 ± 1.62*	7.45 ± 1.33*#	**<0.0001**
Glucose concentration at admission (mg/dL)	503.68 ± 205.55	468.16 ± 239.84	NA	0.6760
pH	7.18 ± 0.09$	7.38± 0.02$	Within normal ranges (7.35–7.45)	**<0.0001** (Welch)
HCO3- (mmol/L)	10.59 ± 3.26$	21.56 ± 1.21$	Within normal ranges (21–27)	**<0.0001** (Welch)
BE (mval/l)	−18.20 ± 4.72$	−3.48 ± 1.79$	Within normal ranges (-2.3 – 2.3	**<0.0001** (UMW)
pCO_2_ (mm Hg)	22.05 ± 7.79$	35.47 ± 4.16$	Within normal ranges (35–45)	**<0.0001** (UMW)
pO_2_ (mm Hg)	72.40 ± 20.47	71.19 ± 14.94	Within normal ranges (75–100)	0.7457 (UMW)
O_2_ sat. (%)	87.57 ± 17.64	91.82 ± 12.36	Within normal ranges (95–98)	0.3282 (UMW)
eGFR (mL/min/1.73m^2^)	83.87 ± 16.80	91.65 ± 28.00	93.26 ± 16.78	0.4044
Na^+^ (mmol/L)	134.38 ± 5.80	133.82 ± 5.24	Within normal ranges	0.8008
Effective serum osmolality (mOsm/kg H_2_O)	296.75 ± 8.41	293.65 ± 8.13	Within normal ranges	0.3435
Hematocrit (%)	41.78 ± 2.96	40.96 ± 2.41	39.81 ± 1.73	0.1553
MCV (fL)	82.90 ± 5.18	82.60 ± 2.27	83.90 ± 2.18	0.7620 (K-W)
DDI (U/kg)	0.91 ± 0.31$	0.59 ± 0.23$	0.81 ± 0.30	**0.0271**
BMI z-score	−0.62 ± 1.08	−0.94 ± 1.52*	−0.42 ± 0.77*	**0.0259**
C-peptide (ng/mL)	0.30 ± 0.24#	0.67 ± 0.45*	0.02 ± 0.02*#	**<0.0001**
Total cholesterol (mg/dL)	154.32 ± 39.77	149.26 ± 27.07	175.25 ± 29.44	0.1072
Triglycerides (mg/dL)	82.17 ± 29.57	70.18 ± 25.12	63.47 ± 16.16	0.1683
HDL (mg/dL)	55.11 ± 13.21	52.59 ± 12.81*	66.65 ± 8.70*	**0.0327**
LDL (mg/dL)	86.34 ± 29.78	85.47 ± 24.68	98.01 ± 28.87	0.5186

* significant difference in post-hoc test between NDM, and EDM, #—significant difference in post-hoc test between DKA, and EDM, $—significant difference in post-hoc test between DKA, and NDM. ANOVA *p*, value test was provided in the table, unless stated otherwise in round bracket: K-W—Kruskal-Wallis test, UMW, Mann-Whitney *U* test, Welch—Welch’s ANOVA, test. BE, base excess; eGFR, estimated glomerular filtration rate; MCV, mean corpuscular volume; DDI, daily dose if insulin; BMI, body mass index; HDL, high density lipoprotein; LDL, low density lipoprotein. Values in boldface are significant at p < 0.05.

### 3.2 Technical Data Processing

The protocol of data filtering in both ionization modes for the DKA batch and the HG batch is shown in Supplementary Figure S2. After technical filtering in the DKA batch, 543 metabolomic features (248 and 295 in positive and negative, respectively) remained, while in the HG batch, 733 features were deemed eligible for further analysis (359 and 374, respectively) (Figure 1B,C).

### 3.3 Metabolite Set Enrichment Analysis

Initially, using all the metabolomic features passing technical data processing, we searched for pathways disturbed by DKA/HG beyond the acute phase of the episodes (Supplementary Tables S3, S4). Thus, we performed the MS peak to Pathway analysis by using the Metaboanalyst tool. By those means, we found that after the DKA episode following pathways were enriched: “xenobiotic metabolism”, “anti-inflammatory metabolites formation from EPA”, “arachidonic acid metabolism”, and “prostaglandin metabolism” and “glycerophospholipid metabolism” were diminished (Figure 1D). After the HG episode, we detected only down-regulated pathways and they were associated with “hormone biosynthesis”, “hexose phosphorylation”, “sphingolipid metabolism”’, and “arachidonic acid metabolism” (Figure 1E).

### 3.4 Selection of Metabolic Features for the Identification Procedure

**FIGURE 2:**
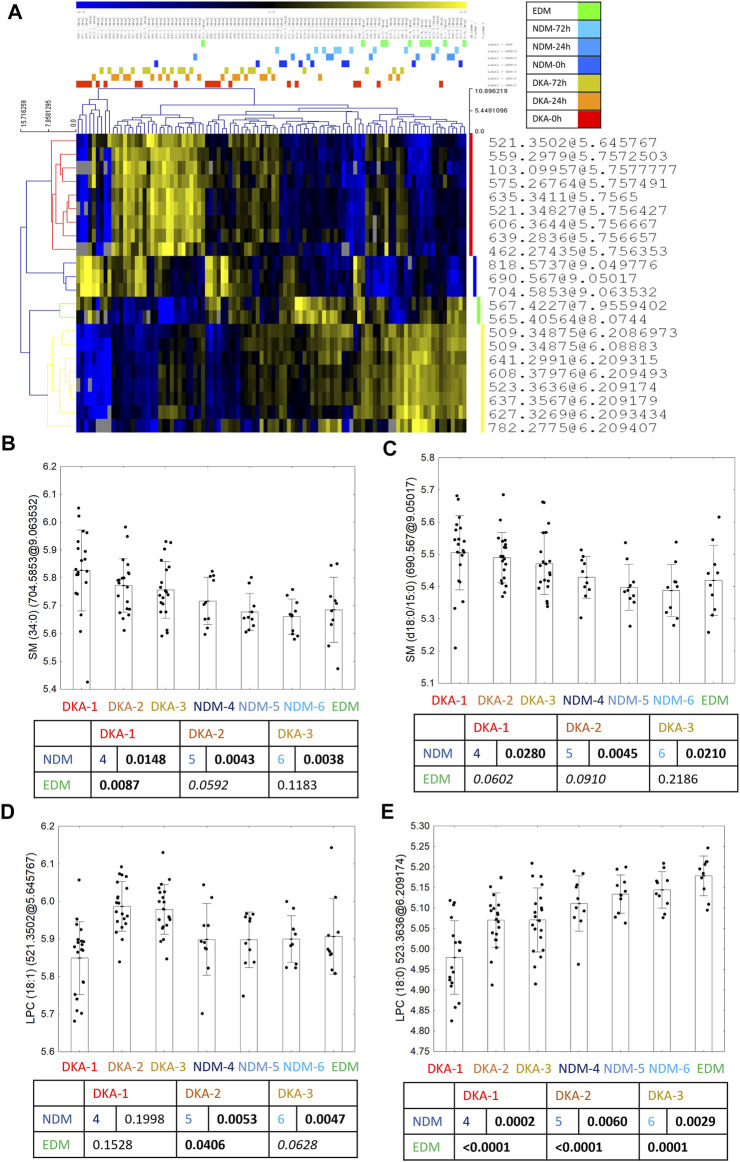
Twenty-two metabolic features selected in the statistical analysis as biomarkers of DKA occurrence **(A)**. Between the group profile of identified metabolites: SM (34:0) **(B)**, SM (d18:0/15:0) **(C)**, LPC (18:1) **(D)** and LPC (18:0) **(E)**. Uncorrected *t*-test *p* values are shown in the graphs.

For the DKA batch, after overlapping results of univariate and advanced statistical modeling (both OPLS-DA models), 22 metabolomic features were selected as the fingerprint of the DKA episodes (Figure 2A). Seven features were successfully identified as four metabolites—two sphingomyelins with a higher level after the DKA episode versus comparative groups: SM (34:0) (Figure 2B) and SM (d18:0/15:0) (Figure 2C) as well as LPC (18:1) (2 features) (Figure 2D) which had a lower level during the episode but higher in the post-episode period. One metabolite had a lower level after DKA—LPC (18:0) (2 features) (Figure 2E). A group-specific time point-paired depiction of the identified metabolite level is shown in Supplementary Figure S3.

**FIGURE 3:**
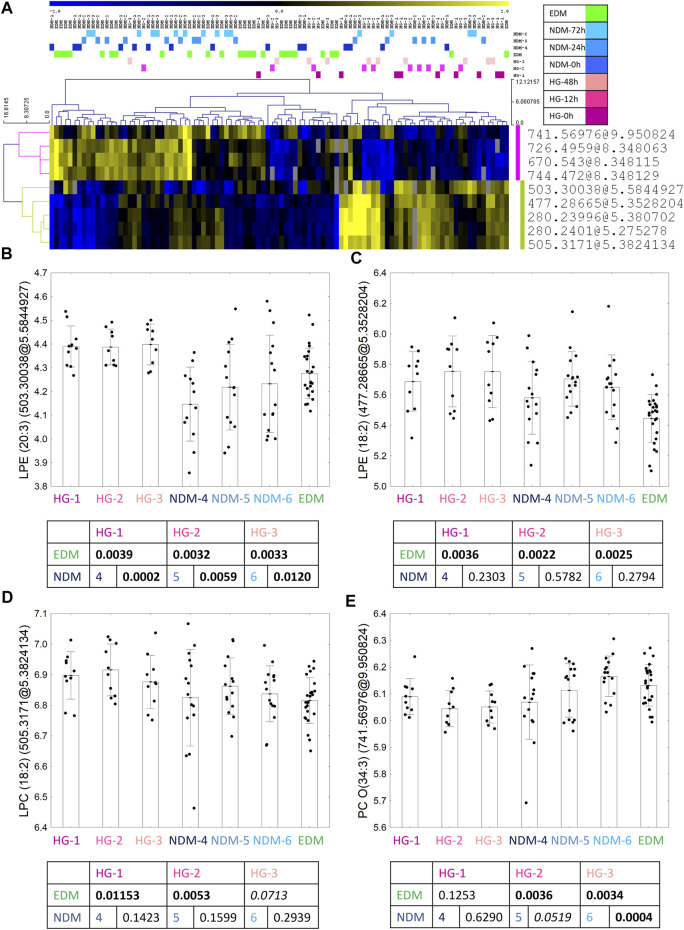
Nine metabolic features selected in the statistical analysis as the biomarkers of HG occurrence **(A)**. Between the group profile of identified metabolites: LPE (20:3) **(B)**, LPE (18:2) **(C)**, LPC (18:2) **(D)** and PC O(34:3) **(E)**. Uncorrected *t*-test *p* values are shown in the graphs.

For the HG batch, after overlapping results of univariate and advanced statistical modeling (both OPLS-DA models), nine metabolomic features were selected as a fingerprint of the HG episodes (Figure 3A). From those, four features were identified as four metabolites—3 with higher after the HG episode versus comparative groups: two lysophosphatidylethanolamine: (LPE) (20:3) (Figure 3B) and LPE (18:2) (Figure 3C) as well as LPC (18:2) (Figure 3D); and one with lower level in HG group - oxy-phosphatidylocholine [PC O-(34:3)] (Figure 3E). A group-specific time point-paired depiction of the identified metabolite level is shown in Supplementary Figure S4.

### 3.5 Correlations Between Identified Metabolites

In the DKA batch, two sphingomyelins strongly correlated with one another (r = 0.94, *p* < 0.0001) (Supplementary Figure S5A) and their correlation with other metabolites: strong positive correlation with LPC (18:0) and a lack of correlation with LPC (18:1) were almost identical (Supplementary Figures S5C–F). Thus, for further analysis, we showed only results for one of them - SM (34:0). SMs’ strong correlations may indicate a similar mechanism leading to their increased level after the DKA episode, however two LPCs: (18:0) and (18:1) poorly correlated with each other (r = 0.18, *p* = 0.0180, Supplementary Figure S5B) suggesting different origins of their serum levels.

In the HG batch, LPC (18:2) and LPE (18:2) were strongly, positively correlated between each other (r = 0.80, *p* < 0.0001, Supplementary Figure S6A), as well as LPE (18:2) and LPE (20:3) (r = 0.49, *p* < 0.0001, Supplementary Figure S6B). Additionally, LPE (20:3) and LPC (18:2) positive, weakly correlated with one another (r = 0.37; *p* = 0.0003, Supplementary Figure S6C). Negative correlations were detected between: PC O-(34:3) and LPE (20:3) (R = -0.36; *p* = 0.0003, Supplementary Figure S6D), PC O-(34:3) and LPE (18:2) (R = -0.24, *p* = 0.0157, Supplementary Figure S6E), and between LPC (18:2) and PC O-(34:3) which was at the borderline of statistical significance (R = -0.17; *p* = 0.0902, Supplementary Figure S6F).

### 3.6 Associations Between Identified Metabolites and Patients’ Clinical Data

**FIGURE 4:**
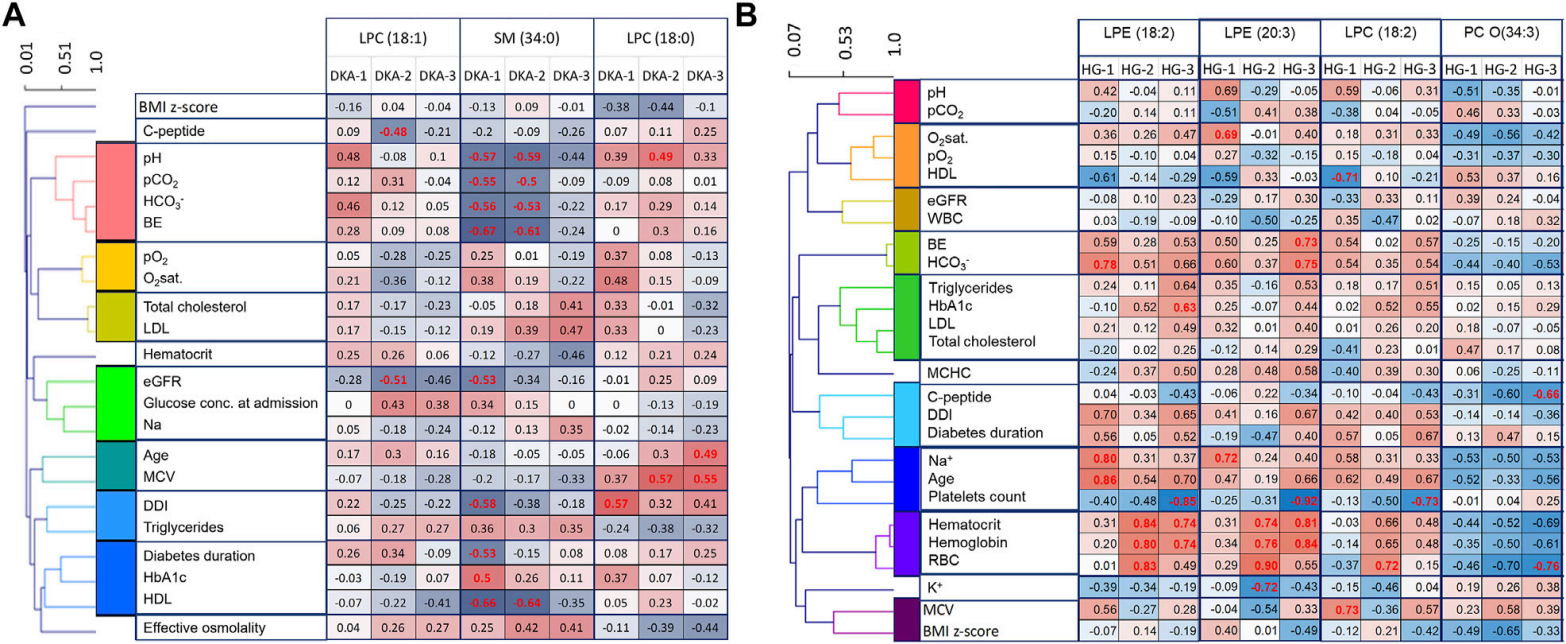
Clinical features correlations with DKA markers **(A)** and for HG markers **(B)**. In order to ease the data interpretation, clinical features were clustered based on Pearson’s correlation coefficient. Significant correlation coefficients are red.

Clinical data were clustered to show common changing patterns and help the interpreting correlations with metabolite levels (Figure 4).

For the DKA markers, during the first 2 days, we found that sphingomyelins correlated strongly and negatively with all four DKA markers measured at admission (pH, pCO_2_, HCO_3_^−^ and BE) (Figure 4A). This indicates that sphingomyelin changes were associated with the depth of ketoacidosis and thus strengthens their role as DKA biomarkers. Additionally, we observed a positive correlation between LPC (18:0) and MPV, which was previously shown to be an indicator of the dehydration level during DKA (Małachowska et al., 2020). Also, a negative correlation between both sphingomyelins and HDL were found statistically significant.

For the HG markers, we noticed that positive markers correlated positively with hematocrit, hemoglobin, and RBC, and the negative marker correlated negatively with them (Figure 4B). Those correlations strengthened over time with the weakest being observed in time point just after the episode. This might indicate that red blood cells might be associated with the distorted metabolism of those metabolites in the days following the episode of HG.

### 3.7 Selected Metabolite’s Ability to Diagnose Past Episodes of HG

To check if the selected metabolites could be used as biomarkers of a past episode of the DKA/HG, ROC curve analysis was performed (Supplementary Figures S7–8). The best biomarker of the past DKA episode (after normalization of the blood–gas test, so at least after 24 h since admission) would be LPC (18:1) as it exceeded 0.8 threshold for AUC for the 48 h time point and almost exceeded it for the 72 h time point (AUC = 0.789). For HG, the best diagnostic criteria in all time points were attributed to LPE (20:3) with all AUC values exceeding 0.8 and reaching 100% sensitivity.

## 4 Discussion

Our study is the first comprehensive, multi-time point metabolic fingerprinting of a post-episode period of acute diabetic complications among children with type 1 diabetes. Our quest to search for unique metabolic changes detectable long after the resolution of the acute pathophysiological state, proved that despite an alignment of typical parameters (pH and glucose), acute diabetic complications exert profound metabolic effects that can be traced in the serum up to 2–3 days after the episode. Thus, we found four metabolites with changed levels up to 72 h since the DKA episode (3 with higher level: LPC (18:1), SM (34:0) and SM (d18:0/15:0); and one with lower level: LPC (18:0)) and four metabolites disturbed by the HG episode up to 48 h (3 with higher level: LPE (18:2), LPE (20:3) and LPC (18:2) and one with lower level: PC O-(34:3).

Metabolomic studies of patients with type 1 diabetes mellitus are scarce and usually involve only comparisons with healthy controls (Balderas et al., 2013; Bervoets et al., 2017). The potential of using the metabolomic approach for studying childhood diabetes was summarized in a 2016 article by Frohnert and Rewers (Frohnert and Rewers, 2016) and additionally, a broad review of type 1 and type 2 diabetes mellitus metabolomic studies was published by Arneth et al. (Arneth et al., 2019). The latter highlighted that type 1 diabetes is associated with an elevated level of LPEs and LPCs which was also shown in this study.

The only available metabolomic studies of acute diabetic complications thus far were carried out on the adult population. The authors used metabolomic profiling to characterized ketosis-prone diabetic patients and found that during DKA, their metabolomic profiles started to resemble patients with type 1 diabetes by having an impaired branched-chain amino acids catabolism and an accelerated fatty acid flux to ketones (Jahoor et al., 2021). A scenario resembling DKA onset was simulated in the study by Dutta et al., in which they characterized metabolomic profile patients with type 1 diabetes after 8 h of insulin withdrawal (Dutta et al., 2012). Among 176 changed metabolites, 1-linoleoylphosphatidylcholine was increased among patients without insulin treatment in comparison to the treated ones. This metabolite might be the same as LPC (18:2) (uncertain position of double bonds) which was found in our study to be increased after the HG episode. As after the HG episode, patients typically experienced reactive hyperglycemia, and an increase of LPC (18:2) might reflect that phenomenon in our study. LPC (18:2) was previously: associated with increased incidence of T2D (among women) and obesity (Bagheri et al., 2018), positively correlated with gait speed (Gonzalez-Freire et al., 2019), and negatively with impaired mitochondrial oxidative capacity among elderly population (Semba et al., 2019); and additionally associated with other pathological states like Alzheimer’s disease (Cui et al., 2014), kidney dysfunction, heart failure (Marcinkiewicz-Siemion et al., 2018), and colorectal cancer (Shen et al., 2017). All these strongly suggest that the decrease of LPC (18:2) is not a specific marker of the aforementioned states but is rather associated with a broader mechanism linking all those pathologies—aging. To answer what is a mechanism of LPC (18:2) increase after the HG episode requires further studies but would be interesting in order to search for mechanisms responsible for long-term HG aftermaths.

Other interesting metabolites found in our study are two sphingomyelins which are negatively correlated with pH during the DKA episode (meaning deeper the acidosis was, higher the SM level was observed). Enzymes responsible for SM decay—sphingomyelinases—have a pH-dependent activity. Neutral sphingomyelinase, commonly present in the cell membrane, has its optimal pH equaling 7.5 (Nyberg et al., 1996). Thus, it is tempting to speculate that due to altered pH, the activity of this enzyme is diminished and that is the cause of elevated SM levels observed in our study. Another possible explanation of increased SM levels would be a release of SMs from the membranous myelin sheath from the nervous system (Kikas et al., 2018). More important than the origin of the elevated level of SMs might be its consequences. SMs have an ability to penetrate the aortic wall and accelerate atherogenesis (Portman and Alexander, 1970) which indicates that the post-episode period of DKA is characterized by a pro-atherogenic serum profile. Interestingly, SMs are the main phospholipids in HDL lipoproteins. It was previously proven that SMs inhibit the activity of LCAT (Lecithin-Cholesterol Acyltransferase) enzyme present in HDL which can produce LPCs (Martínez-Beamonte et al., 2013). Negative correlations between SMs and LPC (18:0) and HDL, might suggest that after DKA, an increased SM level is up taken by HDL where SMs decrease the activity of LCAT and by this contributes to a decreased level of LPA (18:0) after the DKA episode.

Other metabolites found in our study are either unknown for the literature—PC O-(34:3) - or little is known about their biological properties (LPE) thus explaining the sources of their altered levels or evaluation of the biological effect on the patients is currently not possible and requires further studies.

There are some limitations to our study. As this is a prospective, observational study and we do not have pre-episode (baseline) samples from the study group, we cannot rule out the possibility that observed differences are caused solely by the DKA/HG episode rather than a pre-existing or concomitant feature of patients experiencing them. For this, the conduction of animal studies would be necessary in which DKA and HG would be induced to prove a cause-and-effect association. Moreover, due to the unknown nature of the observed changes, it is not possible for us to assess observed changes as harmful—meaning requiring treatment, or as beneficial—meaning being part of a compensatory mechanism that helps restoring normal body functioning after the episodes. Here again, animal experiments would be invaluable to unravel those mechanisms. Due to current technical limitations, it is not possible to measure routinely selected metabolites in clinical practice using a high-throughput method, but we believe that future advancements will enable a cost-effective separation of different LPC, LPE, SMs, and PC species in clinical laboratories. Thus, the development of targeted techniques measuring the levels of the aforementioned metabolites irrespective of patient hydration status would be necessary to use in our results in clinical practice and thoroughly validate our results. Eventually, external validation on a new cohort of patient would be needed before any such test could be used in a clinical scenario. Taking into consideration the aforementioned limitations and available sample size, we suggest that any attempts to explain the mechanistic insights or to generalize the obtained results, should be made with caution.

## 5 Conclusion

The post-episode period of DKA/HG is characterized by an altered metabolism of LPCs, LPEs, SMs, and *p*C. We found eight metabolites that different serum levels may be traced in serum up to 72 h after the DKA episode and up to 48 h for the HG episode. Acute complications of diabetes may cause persistent metabolic disturbances long after pH and glucose levels normalize.

## Data Availability

The original contributions presented in the study are included in the article/Supplementary Material; further inquiries can be directed to the corresponding author.

## Abbreviations

ANOVA, analysis of variance; BO, brain oedema; CART, Classification and Regression Trees; CID, collision-induced dissociation; CV, coefficient of variation; DKA, diabetes ketoacidosis; EDM, established diabetes mellitus; GSEA, gene set enrichment analysis; HDL, high density lipoprotein; HG, hypoglycemia; IS, internal standard; ISPAD, International Society of Pediatric and Adolescent Diabetes; k-NN, k nearest neighbor; LCAT, Acyltransferase; LPE, lysophosphatidylethanolamine; MFE, molecular feature extraction; min, minutes; NDM, newly diagnosed diabetes mellitus without diabetes ketoacidosis; OPLS-DA, orthogonal partial least square discriminant analysis; PC O-(34:3), oxy-phosphatidylocholine (34:3); QA, quality assurance; QC, quality control; RSD, relative standard deviation.
