# Supplementary figures and images for: Multi-Timepoint Metabolic Fingerprinting of a Post-Episode Period of Hypoglycemia and Ketoacidosis Among Children With Type 1 Diabetes
Source: Front Mol Biosci. 2022 Jun 23;9:869116. doi: 10.3389/fmolb.2022.869116 (PMC9259852; doi:10.3389/fmolb.2022.869116)

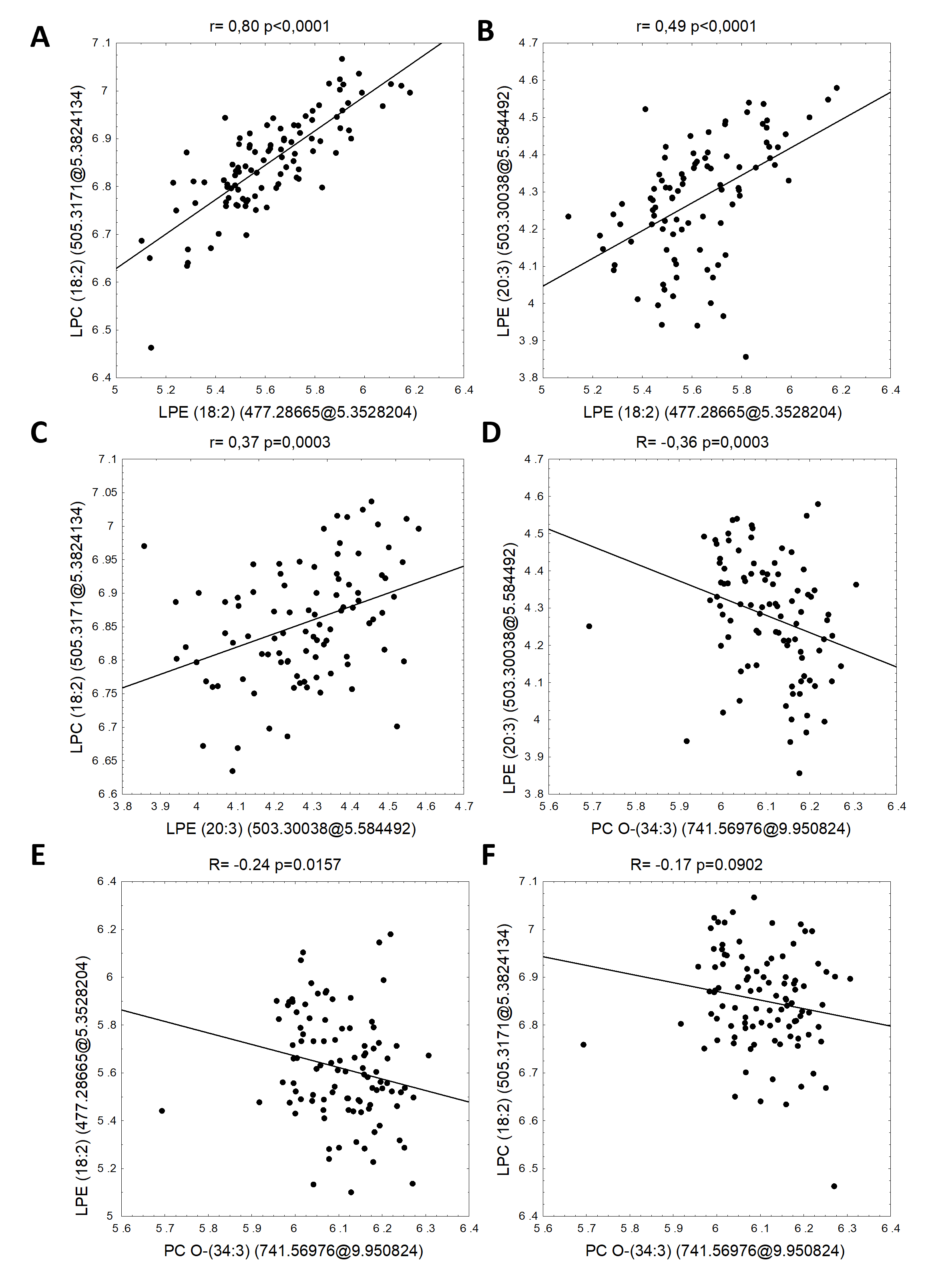

Supplement: Supplementary file 2 [file Image6.TIF]

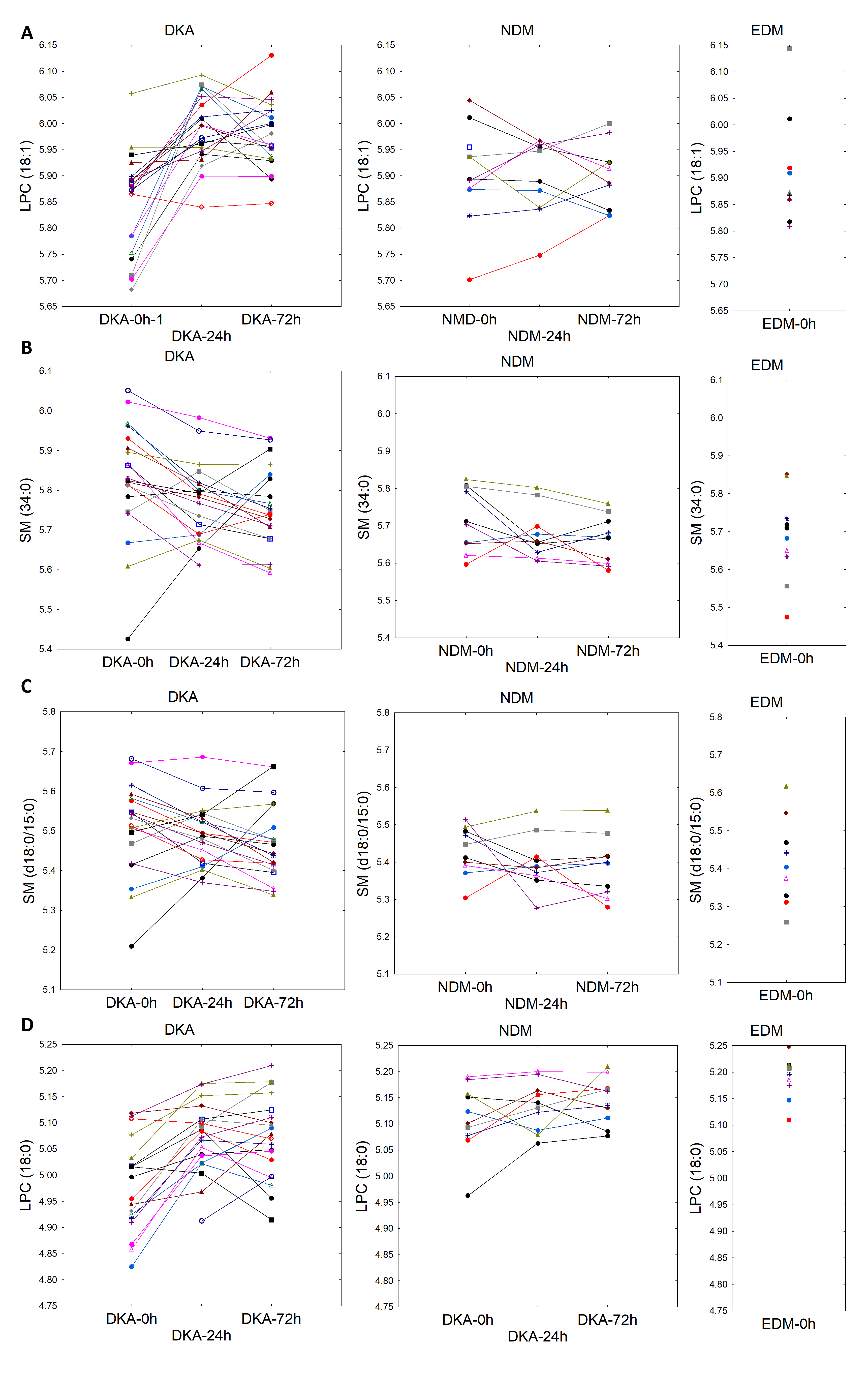

Supplement: Supplementary file 3 [file Image3.TIF]

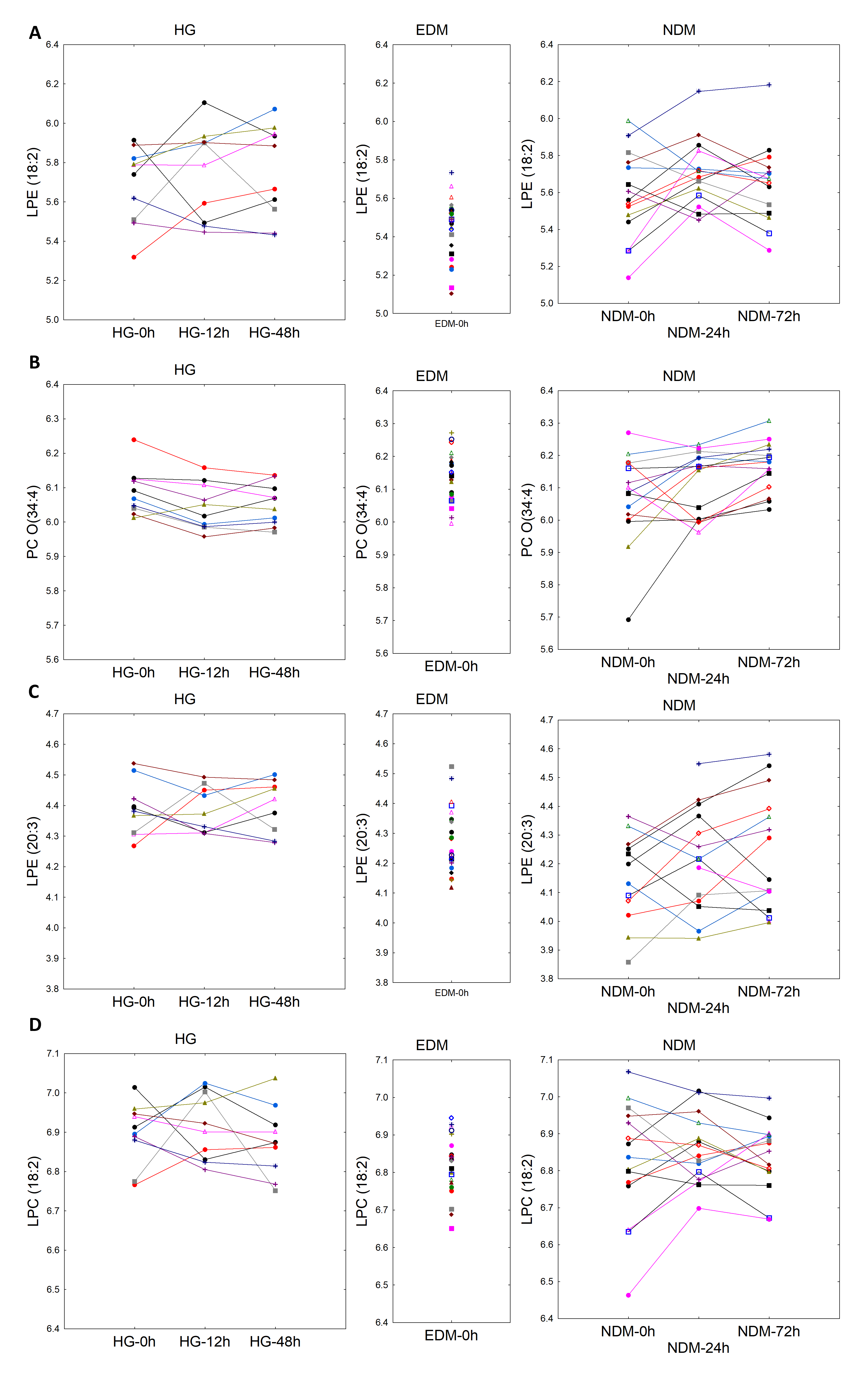

Supplement: Supplementary file 4 [file Image4.TIF]

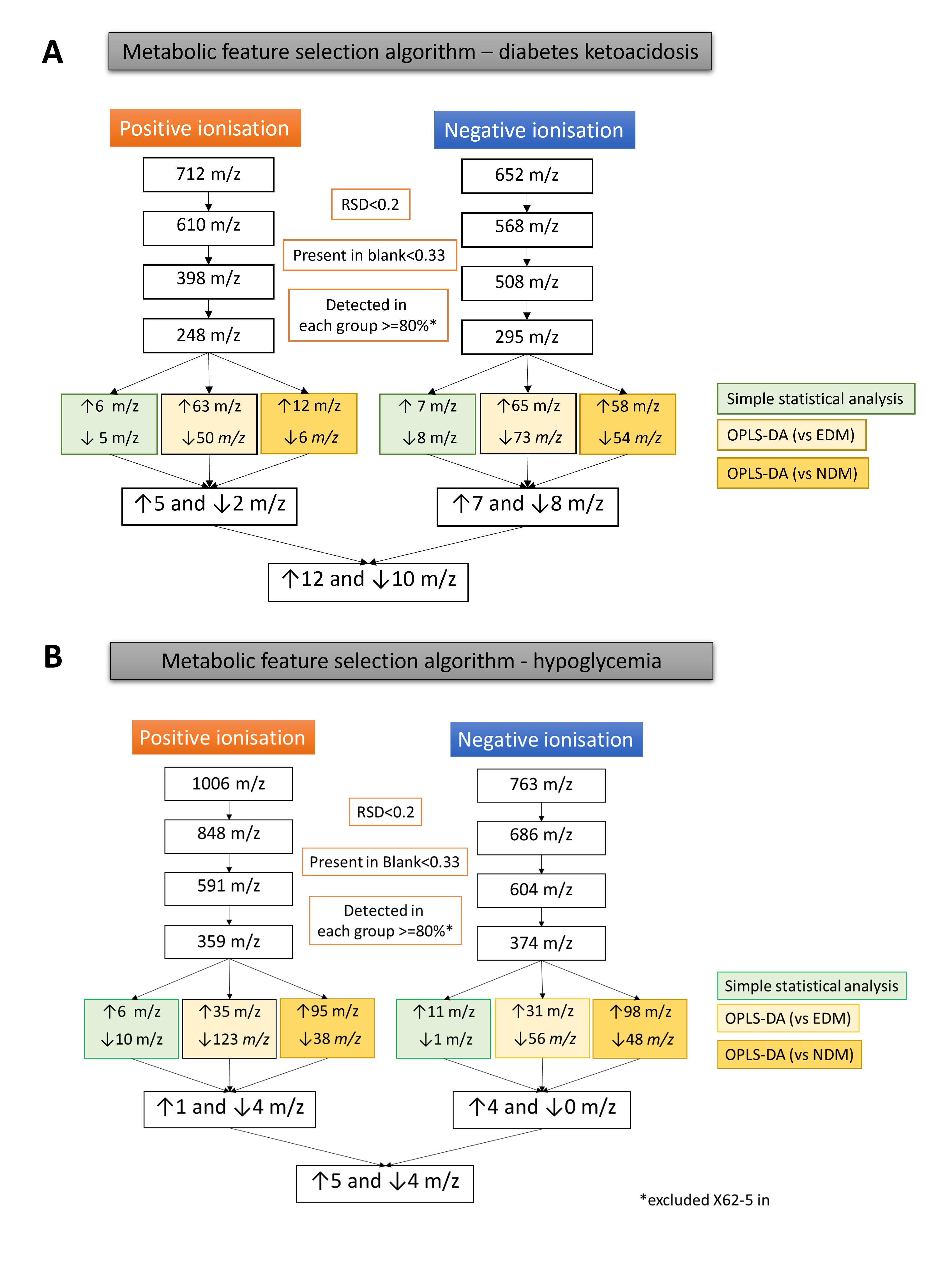

Supplement: Supplementary file 5 [file Image2.TIF]

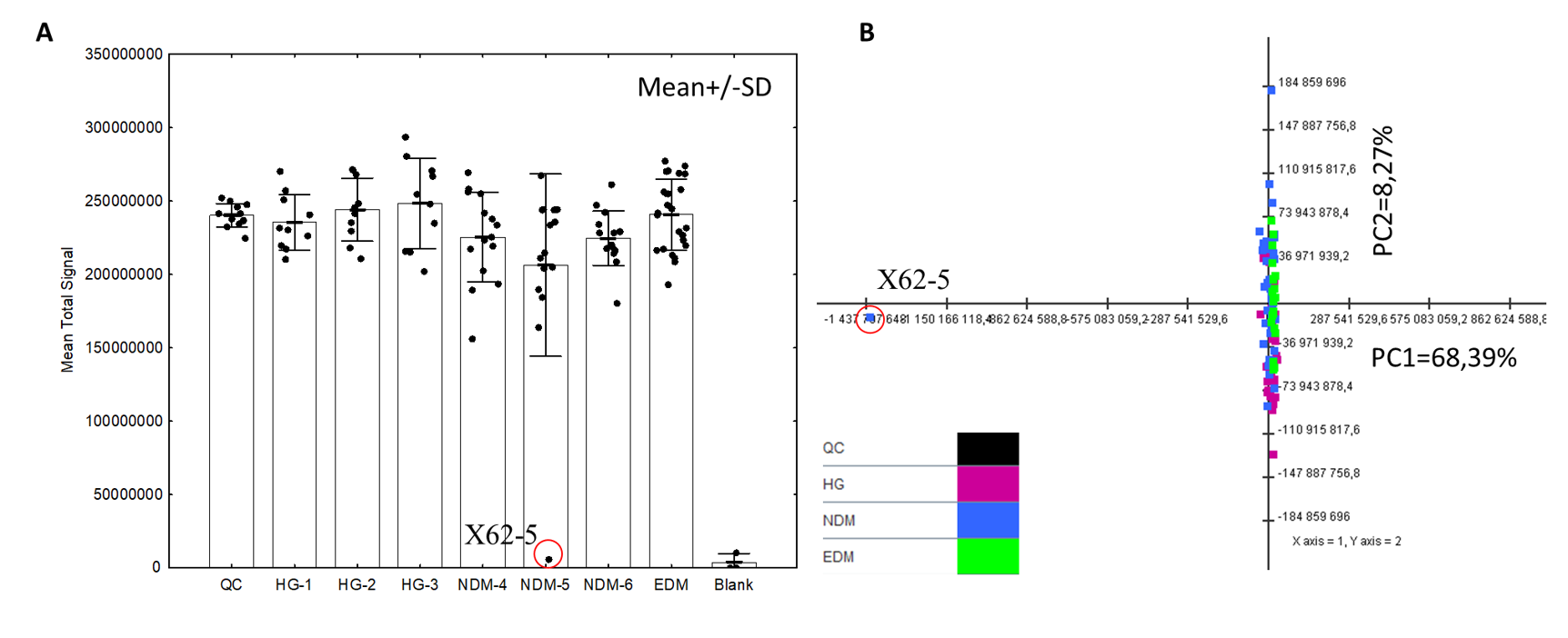

Supplement: Supplementary file 6 [file Image1.TIF]

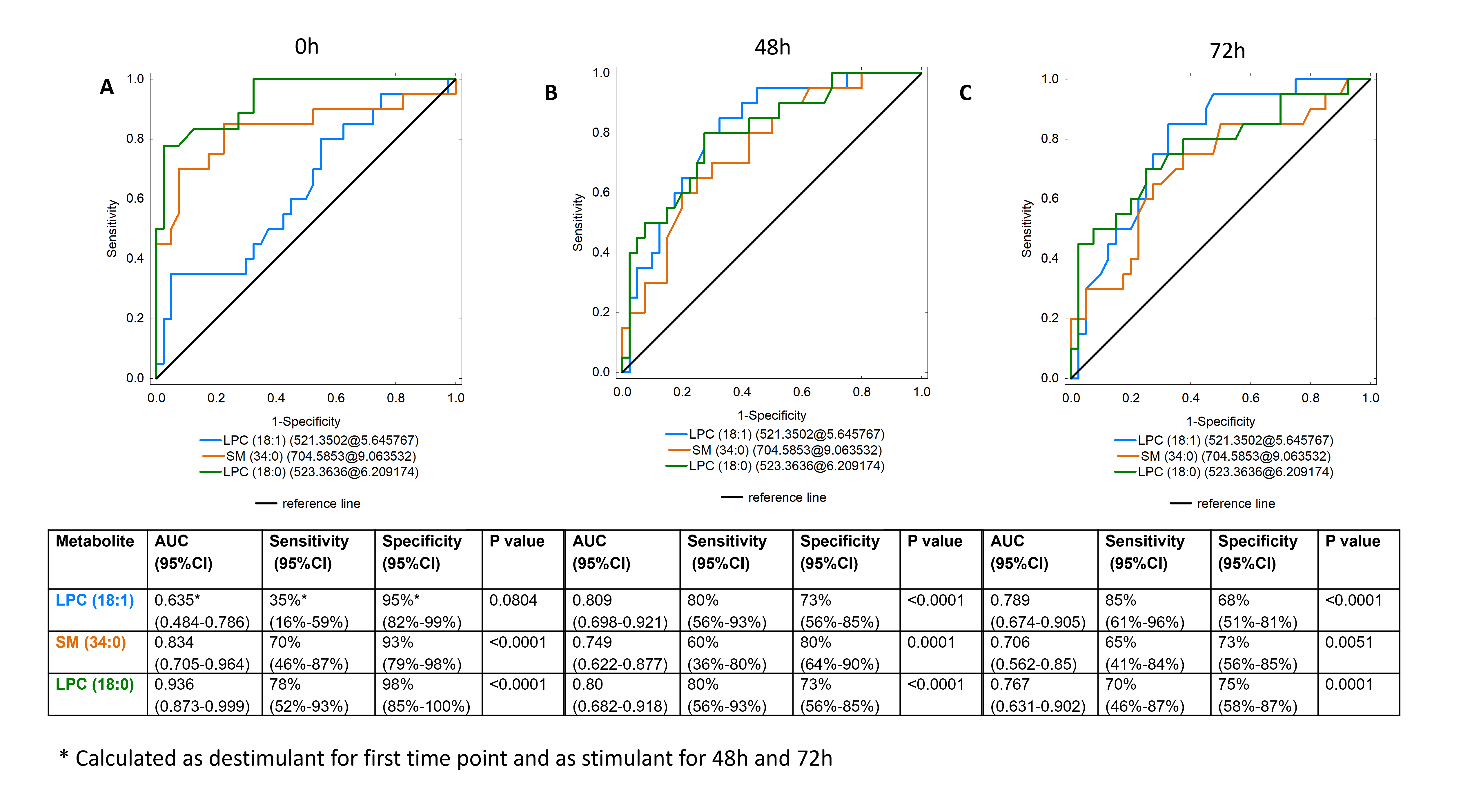

Supplement: Supplementary file 7 [file Image7.TIF]

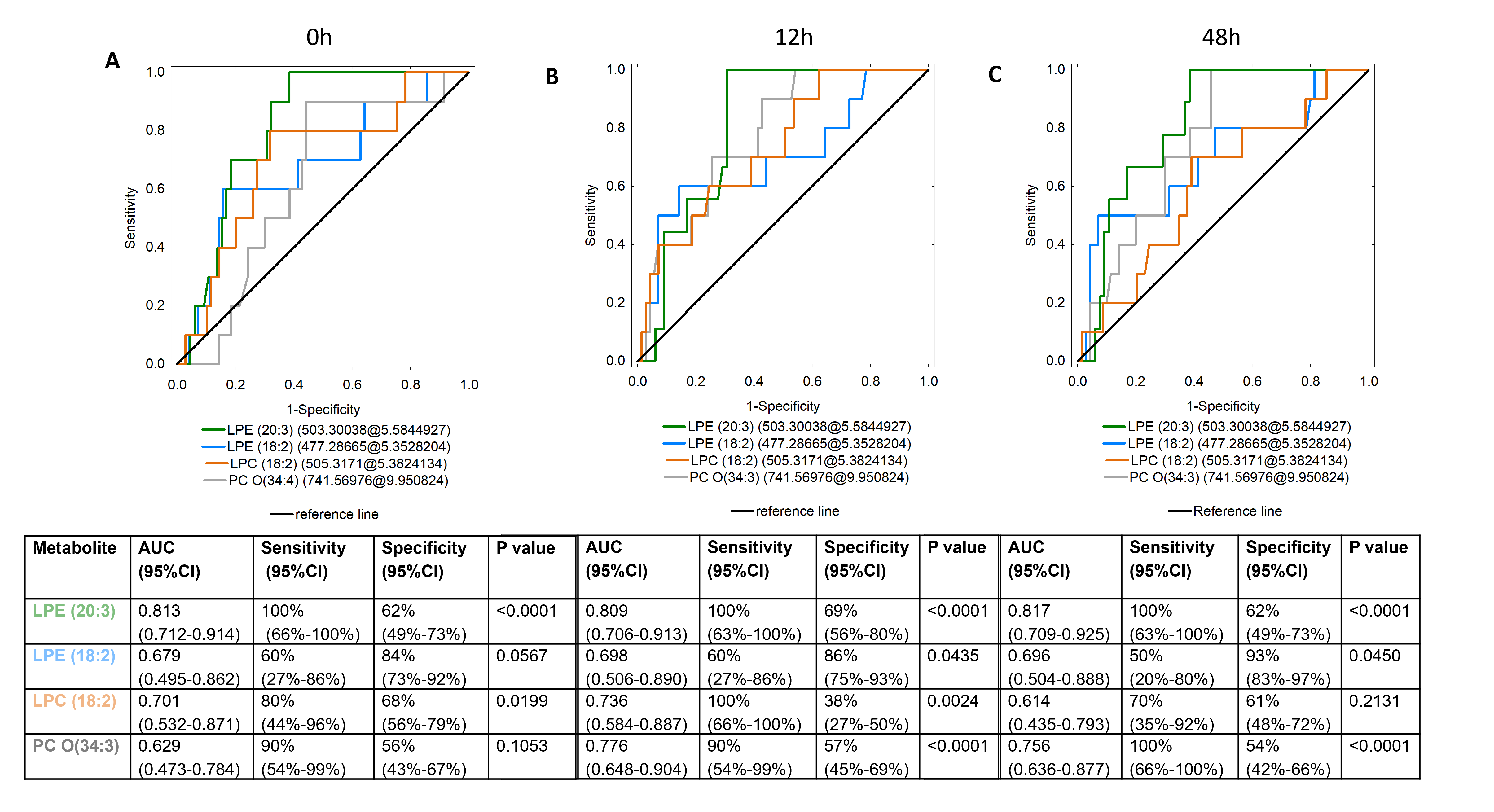

Supplement: Supplementary file 10 [file Image8.TIF]

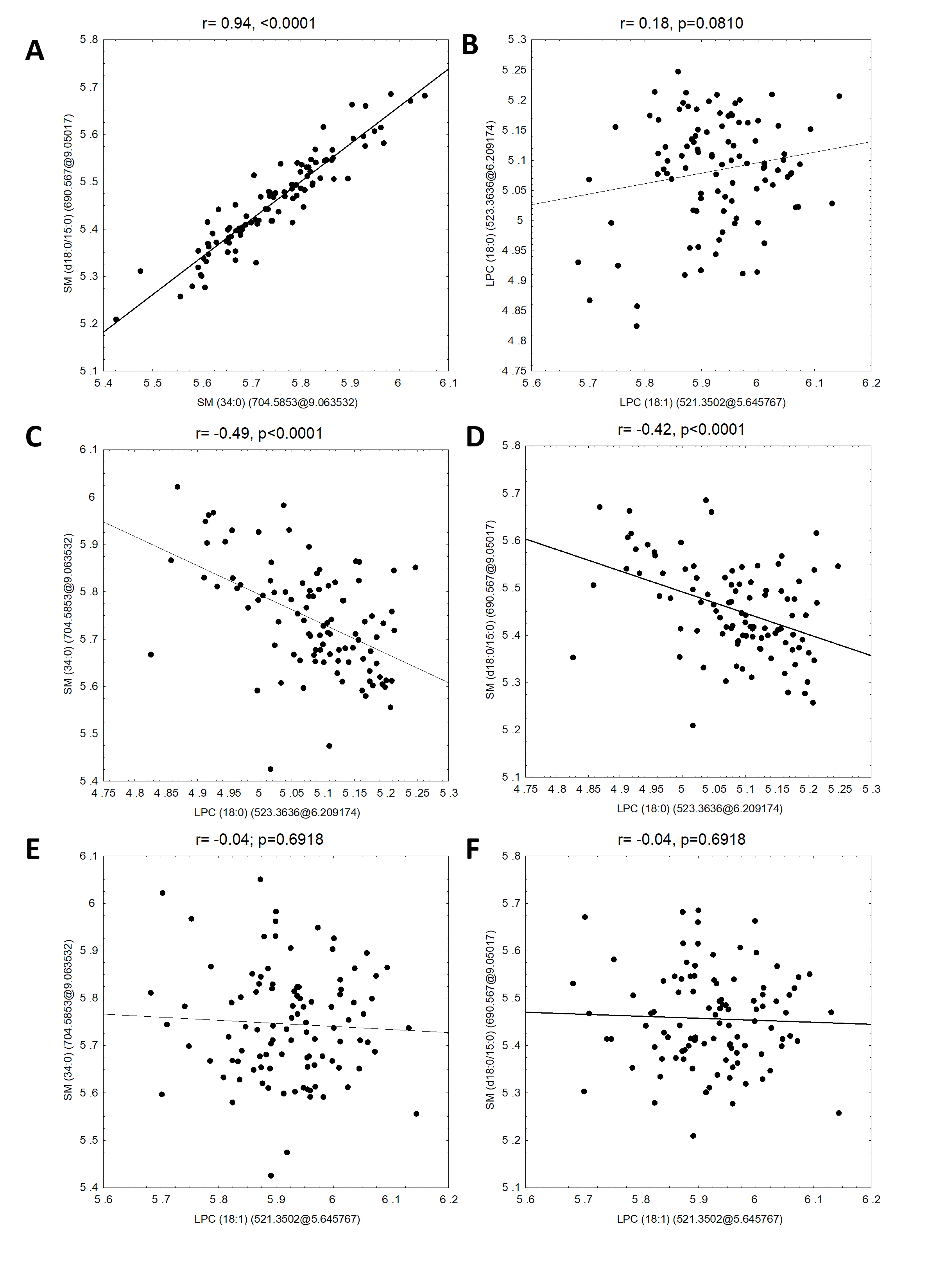

Supplement: Supplementary file 11 [file Image5.TIF]
